# Anti-fibrotic, anti-VEGF or radiotherapy treatments as adjuvants for pterygium excision: a systematic review and network meta-analysis

**DOI:** 10.1186/s12886-017-0601-5

**Published:** 2017-11-25

**Authors:** Wen Zeng, Zengming Liu, Hanjun Dai, Ming Yan, Hong Luo, Min Ke, Xiaojun Cai

**Affiliations:** 1 Department of Ophthalmology, Zhongnan hospital of Wuhan University, No 169 Donghu road, Wuchang District, Wuhan, Hubei 430071 People’s Republic of China; 2 Department of Breast and Thyroid Surgery, Union Hospital, Tongji Medical College, Huazhong University of Science and Technology, Wuhan, People’s Republic of China

**Keywords:** Pterygium, Recurrence, Adjuvant, Mitomycin C, Bevacizumab

## Abstract

**Background:**

Anti-fibrotic, anti-VEGF (vascular endothelial growth factor) medications, or radiotherapy, as adjuvant for pterygium surgical procedure, has been suggested for reducing recurrence, but difficulties may be experienced in deciding which treatment to use. The purpose of this study was to compare the efficacies of these different adjuvants for preventing recurrence following pterygium surgery.

**Methods:**

We conducted a systematic review to identify randomized controlled trials of patients with primary or recurrent pterygium who received anti-fibrotic, anti-VEGF medication, or radiotherapy as adjuvants in combination with surgical procedure. The surgical procedure contained bare sclera technique or petrygium excision combination with tissue grafting. The primary outcome of this study was recurrence. Direct-comparison and Bayesian network meta-analyses were performed to assess direct and indirect evidence of efficacy.

**Results:**

We obtained data from 34 randomized controlled trials, representing a total of 2483 patients. Adjuvants included bevacizumab, 5-FU (5-fluorouracil), MMC (mitomycin C), and β-RT (beta-radiotherapy). Compared with placebo, we found distinguishable improvement in recurrence with bevacizumab (odds ratio [OR] 0.38, 95% confidence interval [CI] 0.18–0.80), MMC (0.12, 95% CI 0.06–0.21), and β-RT (0.17, 95% CI 0.04–0.69), but not with 5-FU (0.41, 95% CI 0.12–1.39). MMC significantly reduced recurrence when compared to bevacizumab (0.31, 95% CI 0.13–0.77) and 5-FU (0.28, 95% CI 0.08–0.99). The probability of having the most recurrences after excision was lowest for MMC, followed by bevacizumab and β-RT. Similar results were found in subgroup analyses, including for primary pterygium, and the patients receiving bare sclera technique or conjunctival autograft.

**Conclusions:**

Adjuvants such as MMC, bevacizumab, and β-RT could effectively prevent recurrence following pterygium excision. However, their efficacy and acceptability require further clarification in future randomized controlled trials.

**Electronic supplementary material:**

The online version of this article (10.1186/s12886-017-0601-5) contains supplementary material, which is available to authorized users.

## Background

Pterygium is an uncontrolled fibrovascular tissue overgrowth of the conjunctiva that overlays the sclera and involves the corneal surface [1]. In addition to cosmetic problems, it could restrict ocular motility, impair visual function, and lead to redness and irritation [2, 3]. Surgical removal may be required when patients want to resolve cosmetic problems, relieve discomfort, improve visual acuity, or plan other ophthalmic surgeries (e.g., for cataracts).

Recurrence after pterygium treatment is a major challenge for both surgeons and patients, as recurrences indicate a greater likelihood of additional recurrences and shorter intervals between recurrences [4]. Surgical removal is an effective technique for pterygium treatment. The bare sclera technique (BST) is the oldest and most basic approach for pterygium; however, the greater recurrence rate after BST, which could be up to 80% [5], has encouraged surgeons to search for more efficient surgical approaches and adjuvant therapies. Tissue grafting, such as with the conjunctival autograft (CA), conjunctival flap, or amniotic membrane transplants (AMTs), can significantly reduce recurrences compared to those occurring with BST alone and have been shown to be superior to BST [6–8]. The combination of the surgical procedure with toxic agents, such as 5-fluorouracil (5-FU), mitomycin C (MMC), and radiotherapy, has also been investigated and has been shown to be effective in preventing recurrences [9–14]. In conjunction with BST, MMC treatment can significantly reduce recurrence compared to that following the use of a placebo [5, 15, 16]. Other studies have also shown a lower recurrence rate following the use of MMC with CAs, conjunctival flap, and AMT, with acceptable complications [17, 18].

More recently, the efficacy and toxicity of anti-VEGF treatment agents such as bevacizumab (avastin) and ranibizumab (lucentis) have been evaluated in primary and recurrent pterygium. Subconjunctival ranibizumab injection in conjunction with pterygium surgery was reported to be well tolerated [19]. Subconjunctival bevacizumab injection without surgery for primary pterygium was shown to be effective for reducing the pterygium size and improving visual function [20, 21]. However, the efficacy of the administration of bevacizumab combined with surgical excision is more controversial [22–27]. Castañeda et al. believed that subconjunctival bevacizumab injection could prevent pterygium recurrences in conjunction with surgery [23]. Ozgurhan et al. found that topical bevacizumab therapy was effective to prevent neovascularization and had lower recurrence rate [27]. However, other studies disagreed with it [24–26]. Treatment efficacy of such antifibrotic or anti-VEGF medications is not currently clear because direct comparisons are rare. Therefore, the goal of this study was to comprehensively review the literature and perform a meta-analysis to determine the relative efficacy of these medications combined with surgical techniques for the treatment of pterygium.

## Methods

### Literature search

This study was performed according to the Preferred Reporting Items for Systematic Reviews and Meta-analyses (PRISMA) statement and the Cochrane Collaboration recommendations [28, 29]. The literature search was performed using PubMed, Ovid MEDLINE, Ovid EM-BASE, and the Web of Science from each database’s inception through September 1, 2015, and updated on March 25, 2016. Search strategy was showed in Additional file 1. Medical Subject Headings together with the search terms (“pterygium” or “pterygia”, “bevacizumab”, “ranibizumab”, “mitomycin C”, “fluorouracil”, “irradiation”) were used. Studies published in English were included in this study, irrespective of publication year or publication type.

The primary objective of this network meta-analysis was to comprehensively review the literature and to determine the relative efficacy of Anti-fibrotic, anti-VEGF medications, or radiotherapy, termed as adjuvants in this study, after surgical excision for primary and recurrent pterygium. Randomized controlled trials comparing the recurrence rates of antifibrotic or anti-VEGF medications to those for placebo after pterygium excision with or without tissue grafting were included in this study. The adjuvants included bevacizumab, MMC, 5-FU, and beta-radiotherapy (β-RT). Nonclinical or non-comparison studies were excluded. The common excision techniques with tissue grafting included CA, limbal CA, conjuntival flaps, and AMT. Since the efficacy of the first three grafts is controversial [30], we combined them into one group, which we called CA.

Two investigators (ZW, LZM) independently reviewed the titles and abstracts of the 153 articles identified by the systematic literature search. If both reviewers agreed that a study did not meet the eligibility criteria, it was excluded. Eighty-four articles received full-text review by a single investigator (ZW), with 30 of the articles randomly selected for independent review by a second author (LZM). All disagreements were settled by the opinion of a third senior reviewer (DHJ).

Recurrence at the last follow-up was the sole outcome measure. No patients were involved in any setting of this study. All data were directly extracted from the article byZW and LZM independently. The extracted data were then double-checked and confirmed by DHJ.

### Risk of bias assessment and evidence grading

Risk of bias was assessed by the Cochrane Collaboration’s tool for the included trials, and three different categories were classified: low risk, high risk, or unclear risk. Study quality was assessed with the Grades of Recommendation, Assessment, Development and Evaluation (GRADE) system, and the quality of evidence was graded into four levels: high, moderate, low, and very low quality.

### Statistical analysis

WinBUGS (version 1.4.3; Medical Research Council Biostatistics Unit, Cambridge, UK) was used to perform a network meta-analysis of recurrence with a random-effects, mixed-treatment comparison model for multiarm trials within the Bayesian framework. A pairwise meta-analysis was conducted by Review Manager (Version 5.0; The Nordic Cochrane Centre, The Cochrane Collaboration, Copenhagen, Denmark). Two-tailed statistical significance was set at 0.05 for hypothesis testing and 0.10 for heterogeneity testing. Two authors (WZ and HJD) independently extracted, checked, and entered the data.

The number of recurrences was used for analysis, and the odds ratio (OR) for recurrence with 95% confidence intervals (CIs) was reported. For the pairwise direct comparison meta-analysis, a random-effects Mantel-Haenszel model was performed for pooled analyses in consideration of heterogeneity. A random-effects Bayesian network meta-analysis with Markov chain Monte Carlo implemented in WinBUGS was used to estimate the treatment effect for each adjuvant. Each adjuvant had at least one common comparator and could be linked to direct and indirect evidence, and the random-effects model allowed the likelihood of statistical heterogeneity between trials. The outcome was assumed as a binomial distribution. To estimate efficacy measures with the Brooks-Gelman-Rubin statistic, a burn-in of 50,000 iterations was used, followed by a further 50,000 iterations for estimation. We used noninformative priors: normal with a mean of 0 and a variance of 10,000 for mean values. The relative probabilities of events in the arms of a study can be parameterized in terms of the logarithm of the OR, and final pooled ORs and their 95% CIs were used to compare treatment effects for outcomes.

The deviance information criterion (DIC) was assessed as a measure of model fit, with lower values meaning a better fit.

## Results

### Description of included studies

**Fig. 1:**
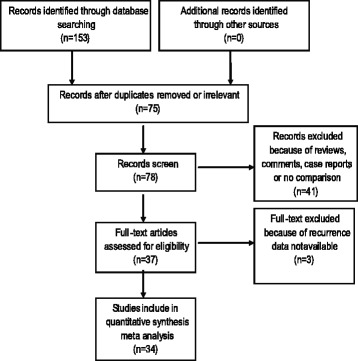
The PRISMA diagram for systematic search and screening process

One hundred fifty-three articles were selected in the electronic search. Seventy-five references were excluded after the title and abstract screen because of irrelevant or duplicate, forty one references were excluded because they were reviews, comments, or case reports and/or had no comparison. Another three references were excluded after full-text review because recurrence data were not available. Finally, thirty-four RCTs were included in this study, including 2483 patients who were randomly assigned to receive placebo (*n* = 749), an anti-proliferation agent, such as MMC (*n* = 1007), 5-FU (*n* = 238), β-RT (*n* = 123), or the anti-VEGF agent bevacizumab (*n* = 366) to prevent recurrence after pterygium surgery. The PRISMA diagram for our systematic search and screening process is presented in Fig. 1.

**Fig. 2:**
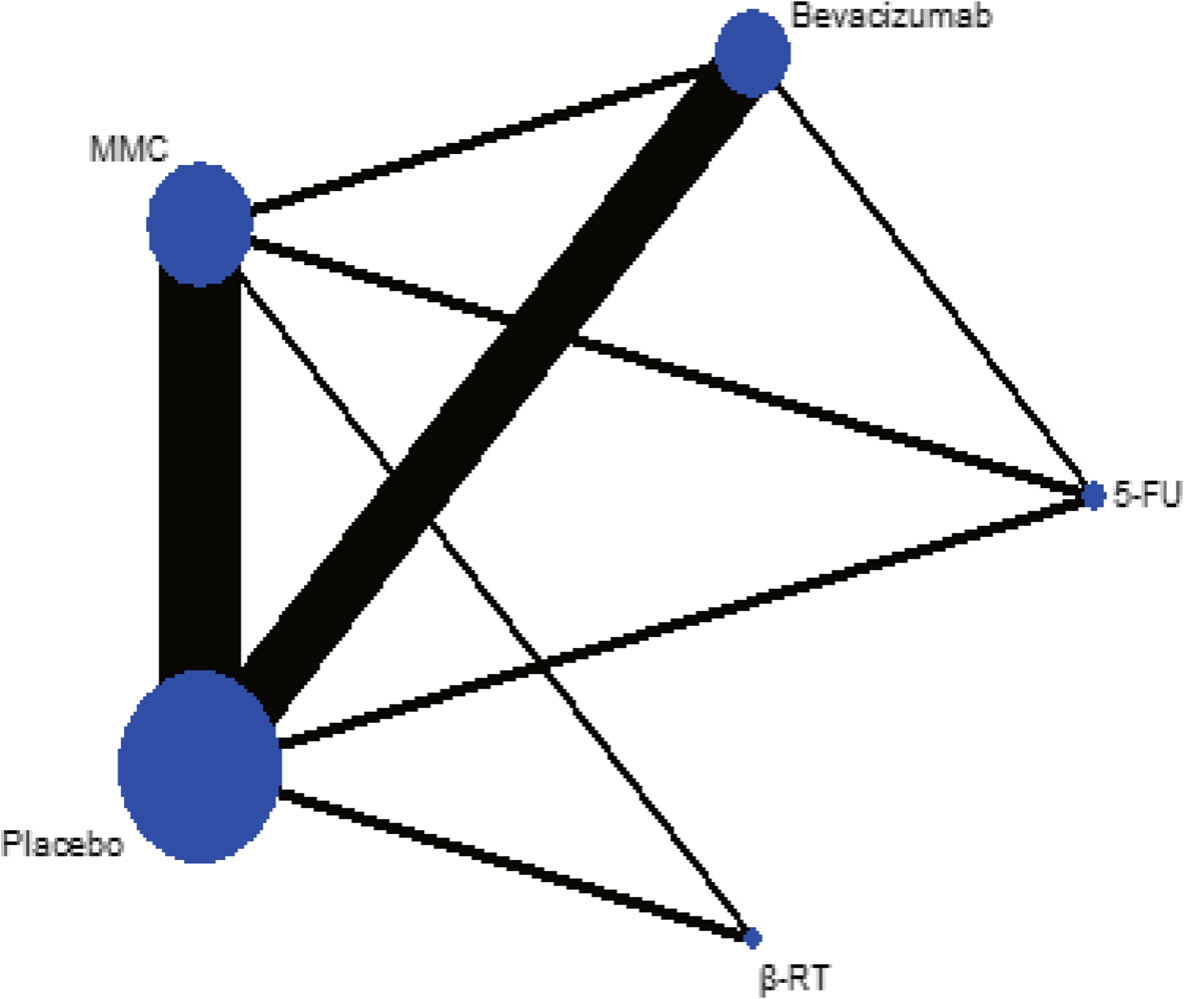
Network of interventional treatments included in meta-analysis. The size of each circles represents the number of studies investigating the drug, the thickness of each connections denotes the total number of samples for the comparison. MMC: mitomycin C, 5-FU: 5-fluorouracil, β-RT: beta-radiotherapy

The diagram for the network of interventional treatments included in the present meta-analysis is shown in Fig. 2. There were 10 trials that compared bevacizumab to placebo [23–27, 31–35], 14 trials that compared MMC to placebo [16, 36–48], 2 that compared 5-FU to placebo [49, 50], 2 that compared β-RT to placebo [51, 52], 2 that compared MMC to 5-FU [53, 54], 1 that compared MMC to β-RT [11], 1 that compared bevacizumab to MMC [55], and 1 that compared bevacizumab with 5-FU [56]. One study compared either bevacizumab or MMC to placebo [57] (Table 1).

**Table 1:** The number of included studies in each comparison group and participants in each treatment group

Comparisons (Treatment1 vs. Treatment2)	Number of Included Studies	No.pts. in Treatment1	No.pts. in Treatment2
Bevacizumab vs. Placebo	11	325	299
MMC vs. Placebo	15	743	384
5-FU vs. Placebo	2	140	140
β-RT vs. Placebo	2	98	96
MMC vs. 5-FU	2	59	71
Bevacizumab vs. MMC	2	45	45
MMC vs. β-RT	1	50	25
5-FU vs. Bevacizumab	1	27	26

*MMC* mitomycin C, *5-FU* 5-fluorouracil, *β-RT* beta-radiotherapy

Baseline features of the included studies are shown in Table 2. Table 2 describes key characteristics of the included studies location, disease codition, excision design, follow-up length, and recurrence definition. Sample sizes ranged from 29 to 240 participants. Studies used different criteria for assessing recurrence: 15 studies used any fibrovascular overgrowth on the cornea; 5 studies used the presence of a fibrovascular overgrowth on the cornea >1.0 mm; 4 studies used fibrovascular overgrowth of >1.5 mm; 1 study used Tseng’s criteria [8]; 1 study evaluated the the recurrence rate of both any fibrovascular overgrowth on the cornea and fibrovascular overgrowth of >1.5 mm; and 8 studies’ recurrence definitions were not stated. The overall quality was rated as low or very low according to the GRADE assessment owing to high risks of bias and heterogeneity. Most studies lacked details about allocation concealment and blinding, and recurrent events among comparisons were less than 300 (Additional file 2).

**Table 2:** Baseline features of the included studies

Study	Location	Disease condition	Excision Design	Arm 1	Arm 2	Arm 3	Mean follow up (month)	Recurrence definition
Castañeda 2014 [23]	Mexico	Primary	CA	Beva	Pla	–	12	I
Karalezli 2014 [24]	Turkey	Primary	CA	Beva	Pla	–	28.9	II
Motarje 2015 [31]	Iran	Primary	BST	Beva	Pla	–	12	I
Ozgurhan 2013 [27]	Turkey	Recurrent	CA	Beva	Pla	–	6	I
Razeghinejad 2010 [25]	Iran	Primary	CA	Beva	Pla	–	7.7	III
Razeghinejad 2013 [26]	Iran	Primary	CA	Beva	Pla	–	6	III
Razeghinejad 2014 [34]	Iran	Primary	CA	Beva	Pla	–	6	I; III
Shahin 2012 [35]	Egypt	Primary	CA	Beva	Pla	–	8	NA
Shenasi 2011 [33]	Iran	Primary	BST	Beva	Pla	–	9	I
Singh 2016 [32]	India	Primary	CA	Beva	Pla	–	3	Tseng’s criteria [8]
Cardillo 1995 [36]	Brazil	No data	CA	MMC	Pla	–	28	II
Junior 2008 [44]	Brazil	Recurrent	CA	MMC	Pla	–	6	I
Ma 2005 [45]	Taiwan	Recurrent	AMT	MMC	Pla	–	28.2	I
Mahar 1993 [16]	Arabia	Primary & Recurrent	BST	MMC	Pla	–	3–36*	I
Mutlu 1999 [41]	Turkey	Recurrent	CA	MMC	Pla	–	15.8	II
Parra 1995 [46]	Spain	Primary	BST	MMC	Pla	–	14.1	I
Pery 2006 [42]	Israel	Primary	CA	MMC	Pla	–	25.3	I
Salman 2010 [43]	Saudi Arabia	Recurrent	AMT	MMC	Pla	–	NA	NA
Chen 1995 [5]	USA	Primary	BST	MMC	Pla	–	11	I
Gupta 2003 [39]	India	Primary & Recurrent	BST	MMC	Pla	–	8.7	II
Lam 1998 [15]	Hong Kong	Primary & Recurrent	BST	MMC	Pla	–	30.1	III
Singh 1990 [48]	USA	Primary & Recurrent	BST	MMC	Pla	–	12.4	NA
Pery 1994 [47]	Israel	Advanced/Recurrent	BST	MMC	Pla	–	8.6	NA
Fakhry 2011 [38]	egypt	Recurrent	CA	MMC	Pla	–	12	I
Silva 2013 [49]	Brazil	No data	CA	5-FU	Pla	–	3	I
Maldonado 1995 [50]	Spain	Primary	BST	5-FU	Pla	–	12.3	NA
Viani 2011 [51]	Brazil	No data	CA	β-RT	Pla	–	18	I
Mourits 2008 [52]	The Netherlands	Primary	BST	β-RT	Pla	–	40	I
M 2013 [55]	Turkey	Primary	CA	Beva	MMC	–	13.85	NA
Ozsutcu 2013 [57]	Turkey	Primary	CA	Beva	MMC	Pla	9	III
Bekibele 2016 [56]	Nigeria	Primary	CA	5-FU	Beva	–	18.35	II
Bekibele 2012 [54]	Nigeria	No data	CA	MMC	5-FU	–	8	I
Kareem 2012 [53]	Iraq	Primary	BST	MMC	5-FU	–	18.8	NA
Pery1993 [11]	Israel	Advanced/Recurrent	BST	MMC	β-RT	–	15.3	NA

*CA* conjunctival autograft transplantation, *BST* bare sclera technique, *AMT* amniotic membrane transplants, *Beva* bevacizumab, *MMC* mitomycin C, *5-FU* 5-fluorouracil, *β-RT* beta-radiotherapy, *Pla* placebo, *, range

Recurrence definition: I: Any fibrovascular overgrowth on the cornea, II: >1.0 mm fibrovascular overgrowth on the cornea, III: >1.5 mm fibrovascular overgrowth on the cornea, NA: not available

### Direct meta-analysis

Pairwise direct-analysis ORs for recurrence are presented in Additional file 3. When compared with placebo, both bevacizumab and MMC significantly benefited patients in terms of recurrence after surgery, with ORs of 0.45 (95% CI, 0.25–0.83) and 0.15 (95% CI, 0.07–0.31), respectively; β-RT had marginal clinical efficacy, with an OR of 0.12 (0.01–1.03). Other direct comparisons, including 5-FU versus placebo, MMC versus 5-FU, bevacizumab versus MMC, bevacizumab versus 5-FU, or MMC versus β-RT, found no significant difference.

### Bayesian network meta-analysis

Both random- and fixed-effects Bayesian models were used, and the deviance information criterion was 316.2 compared to 366.4 (lower values mean a better fit), respectively, indicating a better fit of the former. Results from the random-effects Bayesian network meta-analysis were shown in Table 3, which suggested that, compared with placebo, bevacizumab, MMC, and β-RT reduced the odds of recurrence by 62%, 88%, and 83% (ORs of 0.38 [95% CI, 0.18–0.80], 0.12 [95% CI,0.06–0.21], and 0.17 [95% CI, 0.04–0.69]), respectively, whereas there was no evidence that 5-FU provided a significantly protective effect against the recurrence of pterygium after surgery (OR 0.41; 95% CI, 0.12–1.39). MMC reduced the odds of recurrence by 69% (OR, 0.31; 95% CI, 0.13–0.77) and 72% (OR, 0.28; 95% CI, 0.08–0.99) when compared with bevacizumab and 5-FU, respectively. No other pairs differed significantly. Point estimates from traditional binary meta-analysis were near those from the Bayesian network analysis, and their credible intervals generally overlapped with similar direction, indicating no significant differences between the results of direct and indirect evidence. The probability of having the most recurrences after excision was lowest for MMC (probability = 0), followed by bevacizumab (probability = 1.44) and β-RT (probability = 1.86).

**Table 3:** Bayesian network meta-analysis of recurrence for comparisons

Comparator	Effect Sizes(95% CrI), by Treatment				Probability of being ranked most recurrence (%, 95% CI)	Rank most recurrence
	Bevacizumab	MMC	5-FU	β-RT		
Placebo	0.38(0.18, 0.80)*	0.12(0.06, 0.21)*	0.41(0.12, 1.39)	0.17(0.04, 0.69)*	85.63 (0, 100)	1
Bevacizumab	–	0.31(0.13, 0.77)*	1.1(0.28, 4.30)	0.45(0.10, 2.12)	1.44 (0, 0)	3
MMC	–	–	3.56(1.01, 12.29)*	1.47(0.34, 6.21)	0 (0, 0)	5
5-FU	–	–	–	0.41(0.07, 2.56)	11.07 (0, 100)	2
β-RT	–	–	–	–	1.86 (0, 0)	4

*MMC* mitomycin C, *5-FU* 5-fluorouracil, *β-RT* beta-radiotherapy, *CrI* credible interval, *CI* confidence intervals

*p < 0.05. Effect sizes favor the above (column heading) intervention in each comparison when OR less than 1

### Subgroup Bayesian network meta-analysis

A sensitivity analysis for the recurrence outcome was performed with a subgroup network meta-analysis and was conducted in groups BST, CA, and primary pterygium (Table 4). In primary pterygium, results indicated favorable effects of bevacizumab (OR, 0.41; 95% CI, 0.21–0.78), MMC (OR, 0.08; 95% CI, 0.03–0.19), and β-RT (OR, 0.10; 95% CI, 0.02–0.40). For conjunctival autografts, network meta-analysis significantly favored MMC (OR, 0.23; 95% CI, 0.10, 0.51) and 5-FU (OR, 0.19; 95% CI, 0.05, 0.78) over placebo. In BST, results indicated significant differences for MMC (OR, 0.05; 95% CI, 0.014–0.10) and β-RT (OR, 0.17; 95% CI, 0.01–0.42), respectively, compared with placebo, and MMC with 5-FU (OR, 0.01; 95% CI, 0.006, 0.28), which all favored the formers. In the above-mentioned three subgroup analyses, the rank probabilities all suggested that MMC treatment was likely to have the lowest recurrence, which was similar to the results from total pterygium analyses (Table 3).

**Table 4:** Subgroup-analysis of recurrence for comparisons

Subgroup analysis	Comparator	Effect Sizes(95% CrI), by Treatment				Probability of being ranked most recurrence (%, 95% CI)	Rank most recurrence
		Bevacizumab	MMC	5-FU	β-RT		
Primary	Placebo	0.41 (0.21, 0.78)^*^	0.08 (0.03, 0.19)^*^	1.01 (0.26, 4.12)	0.10 (0.02, 0.40)^*^	45.45 (0, 100)	2
	Bevacizumab	–	0.20 (0.07, 0.51)^*^	2.61(0.59, 10.80)	0.23 (0.05, 1.10)	0.62 (0, 0)	3
	MMC	–	–	13.13 (3.01, 56.51)^*^	1.17 (0.28, 5.38)	0 (0, 0)	5
	5-FU	–	–	–	0.01 (0.09, 0.66)^*^	53.47 (0, 100)	1
	β-RT	–	–	–	–	0.47 (0, 0)	4
CA	Placebo	0.50 (0.23, 1.02)	0.23 (0.10, 0.51)^*^	0.19 (0.05, 0.78)^*^	0.33 (0.04, 2.31)	77.06 (0, 100)	1
	Bevacizumab	–	0.45 (0.17, 1.27)	0.39 (0.09, 1.76)	0.67 (0.08, 5.69)	4.44 (0, 0)	2
	MMC	–	–	0.87 (0.18, 3.82)	1.47 (0.17, 12.33)	0.21 (0, 0)	3
	5-FU	–	–	–	1.70 (0.15, 19.32)	2.37 (0, 0)	2
	β-RT	–	–	–	–	15.91 (0, 100)	1
BST	Placebo	0.46 (0.042, 1.16)	0.05 (0.014, 0.10)^*^	2.26 (0.14, 6.11)	0.17 (0.01, 0.42)^*^	46.92 (0, 100)	2
	Bevacizumab	–	0.44 (0.02, 1.13)	595.9 (0.33, 50.65)	2.22 (0.027, 3.74)	4.30 (0, 0)	3
	MMC	–	–	98.73 (3.54, 167.50)^*^	4.72 (0.31, 11.60)	0.002 (0, 0)	5
	5-FU	–	–	–	0.84 (0.006, 1.003)	47.96 (0, 100)	1
	β-RT	–	–	–	–	0.81 (0, 0)	4

*MMC* mitomycin C, *5-FU* 5-fluorouracil, *β-RT* beta-radiotherapy, *CrI* credible interval, *CI* confidence intervals

*p < 0.05. Effect sizes favor the above (column heading) intervention in each comparison when OR less than 1

## Discussions

Recurrence after BST, which is associated with more ocular morbidity and the risk of recurrence, has been a major challenge for pterygium treatment. Tissue grafting and adjuvant treatments have been developed to prevent recurrence and are currently commonly used in pterygium excision. Studies have indicated that recurrence was decreased when combining adjuvants with tissues grafting [9–14]. The use of anti-VEGF medications, such as bevacizumab, with improvement of recurrence, deserves a fresh assessment of published evidence for the adjuvants for pterygium surgery. For pterygium, the RCTs are rare, relatively small, and lacking all of the simultaneous intervention comparisons. Network meta-analysis is thus the optimal tool for evaluating the efficacy of these adjuvant agents in pterygium, considering its advantage in pooling data from both direct and indirect comparisons.

To our knowledge, the present study is the first network meta-analysis in this field. Results from the present study found out that, in both pairwise direct comparisons and Bayesian network meta-analyses, MMC, β-RT, and bevacizumab, but not 5-FU, were significantly more effective than was placebo for reducing recurrence following pterygium excision with or without tissue grafting. MMC had the highest probability of having the most efficacy in reducing recurrence. Although bevacizumab ranked higher than β-RT for efficacy, this difference was not statistically significant. Results from subgroup analyses, including primary pterygium and pterygium excision with BST or CA, were consistent with these data. Data for recurrent pterygium and pterygium excision with AMT were insufficient, and no such analyses were conducted in this study.

Despite the valuable data gathered on this subject, several aspects remain to be considered in future studies. For instance, the use of adjuvants made the surgical procedures more complicated and added economic burden, especially bevacizumab. Analogously, side effects associated with adjuvant administration also challenges the benefit-risk ratio. Such issues might decrease the acceptability of adjuvants. Therefore, the selection of suitable candidates who will benefit the most from adjuvant treatments may be an important issue. Thus, it is important to determine risk factors for recurrence. Some studies reported that age, morphology, and increased inflammation after surgery might contribute to a greater risk of recurrence after surgery [58–64]. However, recurrence risk assessment tools for routine clinical practice have been neglected in pterygium.

In addition, the optimal dosage, duration, and administration for adjuvants are unclear. The dose of β-RT is 25 Gy with BST [52], which is decreased to 10 Gy when it is combined with CA [51]. The dosage and administration of 5-FU were consistent among trials, but not the duration, which varied from 3 to 5 min. For bevacizumab, the dosage varied from 1.25 mg to 7.5 mg, and the administration approach can be a subconjunctival injection or eye drops. The administration procedure for MMC is more complicated, with different dosages, durations, and approaches. Many studies have addressed this need for standardization, but unfortunately, no routine practice has yet been recommended.

Finally, our findings must be taken in light of several limitations. First, the number of recurrent events in our study was small (472 recurrences), leading to an imprecise point assessment, which might increase the possibility of a Type II statistical error. Second, despite the effort taken to ensure a full search of the literature, missing reports might have affected the calculated estimates of the treatment effects, especially as trials in this field were rare and small in size. Finally, the follow-up duration in some studies included in the present review might not have been sufficient. Reports showed that 50% of recurrences might occur with in 4 months, and 97% might occur within 12 months [62, 63]. Thus, follow-up of at least 1 year is appropriate. The mean follow-up duration in 13 studies was less than 1 year, which might have affected the calculated estimates of the treatment effects. Finally, the recurrence definition varied among studies. According to a 4-point scale [2], termed Tseng’s criteria, a true corneal recurrence is defined when a fibrovascular tissue invades the cornea, termed as Grade 4. The recurrence definition for trials included in the present study reached Grade 4 recurrence but more intensive: any, >1.0 mm and >1.5 mm fibrovascular overgrowth on the cornea. A standardized definition for pterygium recurrence is warranted when RCTs are conducted.

## Conclusion

Collectively, with RCT-based evidence, our study results suggest that bevacizumab, MMC, and β-RT are likely to reduce recurrence compared with placebo, and MMC might have the lowest risk of recurrence as an adjuvant agent for pterygium after surgery. However, the efficacy and acceptability of these adjuvants still needs to be determined.

## Additional files

Additional file 1: Search strategy (DOC 24 kb)

Additional file 2: Quality assessment for included trials (PDF 513 kb)

Additional file 3: Forest plots from the direct meta-analysis of recurrence between comparisons. Data are presented with odds ratios and 95% confidence intervals (CI) (PDF 385 kb)

Additional file 4: Original recurrence data of this study (XLS 18 kb)

## Abbreviations

5-FU: 5-fluorouracil
AMTs: Amniotic membrane transplants
BST: Bare sclera technique
CA: Conjunctival autograft
CIs: Confidence intervals
DIC: Deviance information criterion
MMC: Mitomycin C
OR: Odds ratio
β-RT: Beta-radiotherapy
