# Supplementary material for: Anti-fibrotic, anti-VEGF or radiotherapy treatments as adjuvants for pterygium excision: a systematic review and network meta-analysis
Source: BMC Ophthalmol. 2017 Nov 25;17:211. doi: 10.1186/s12886-017-0601-5 (PMC5702200; doi:10.1186/s12886-017-0601-5)
Supplement: Supplementary file 1 — Search strategy (DOC 24 kb) [file 12886_2017_601_MOESM1_ESM.doc]

**File S1. Search strategy.**

The search strategy for Pubmed was as the following:

#1 pterygium[Title/Abstract] OR pterygia[Title/Abstract]

#2 bevacizumab[Title/Abstract] OR avastin[Title/Abstract]

#3 ranibizumab [Title/Abstract] OR lucentis[Title/Abstract]

#4 mitomycin C[Title/Abstract] OR MMC[Title/Abstract]

#5 fluorouracil[Title/Abstract] OR 5-FU[Title/Abstract]

#6 radiotherapy[Title/Abstract] OR β-irradiation[Title/Abstract] OR β-RT[Title/Abstract]

#7 #1 AND #2

#8 #1 AND #3

#9 #1 AND #4

#10 #1 AND #5

#11 #1 AND #6
