# Supplementary material for: Anti-fibrotic, anti-VEGF or radiotherapy treatments as adjuvants for pterygium excision: a systematic review and network meta-analysis
Source: BMC Ophthalmol. 2017 Nov 25;17:211. doi: 10.1186/s12886-017-0601-5 (PMC5702200; doi:10.1186/s12886-017-0601-5)
Supplement: Supplementary file 2 — Quality assessment for included trials (PDF 513 kb) [file 12886_2017_601_MOESM2_ESM.pdf]

A

Bevacizumab for pterygium excision

Patient or population: patients with pterygium excision
Settings:
Intervention: Bevacizumab

Outcomes	Illustrative comparative risks* (95% CI)		Relative effect (95% CI)	No of Participants (studies)	Quality of the evidence (GRADE)	Comments
	Assumed risk Control	Corresponding risk Bevacizumab				
**Recurrence**	**Study population**		**OR 0.45** (0.25 to 0.83)	624 (10 studies)	⊕⊕⊖⊖ **low**[1,2,3]	
	**231 per 1000**	**119 per 1000** (70 to 199)				
	**Moderate**					
	**133 per 1000**	**65 per 1000** (37 to 113)				

*The basis for the **assumed risk** (e.g. the median control group risk across studies) is provided in footnotes. The **corresponding risk** (and its 95% confidence interval) is based on the assumed risk in the comparison group and the **relative effect** of the intervention (and its 95% CI).

CI: Confidence interval; **OR:** Odds ratio;

GRADE Working Group grades of evidence
High quality: Further research is very unlikely to change our confidence in the estimate of effect.
Moderate quality: Further research is likely to have an important impact on our confidence in the estimate of effect and may change the estimate.
Low quality: Further research is very likely to have an important impact on our confidence in the estimate of effect and is likely to change the estimate.
Very low quality: We are very uncertain about the estimate.

1 Heterogeneity: $Tau^2 = 0.31$; $Chi^2 = 14.58$, df = 10 (P = 0.15); $I^2 = 31\%$
2 Event=108, less than 300
3 Studies lacked details about allocation concealment and blinding

B

MMC for pterygium excision

Patient or population: patients with pterygium excision
Settings:
Intervention: MMC

Outcomes	Illustrative comparative risks* (95% CI)		Relative effect (95% CI)	No of Participants (studies)	Quality of the evidence (GRADE)	Comments
	Assumed risk Control	Corresponding risk MMC				
**Recurrence**	**Study population**		**OR 0.15** (0.07 to 0.31)	1127 (15 studies)	⊕⊕⊖⊖ **low**[1,2,3]	
	**396 per 1000**	**89 per 1000** (44 to 169)				
	**Moderate**					
	**389 per 1000**	**87 per 1000** (43 to 165)				

*The basis for the **assumed risk** (e.g. the median control group risk across studies) is provided in footnotes. The **corresponding risk** (and its 95% confidence interval) is based on the assumed risk in the comparison group and the **relative effect** of the intervention (and its 95% CI).

CI: Confidence interval; **OR:** Odds ratio;

GRADE Working Group grades of evidence
High quality: Further research is very unlikely to change our confidence in the estimate of effect.
Moderate quality: Further research is likely to have an important impact on our confidence in the estimate of effect and may change the estimate.
Low quality: Further research is very likely to have an important impact on our confidence in the estimate of effect and is likely to change the estimate.
Very low quality: We are very uncertain about the estimate.

1 Heterogeneity: $Tau^2 = 1.36$; $Chi^2 = 46.79$, df = 14 (P < 0.0001); $I^2 = 70\%$
2 Studies lacked details about allocation concealment and blinding
3 Event=238, less than 300

C

5-FU for pterygium excision

Patient or population: patients with pterygium excision
Settings:
Intervention: 5-FU

Outcomes	Illustrative comparative risks* (95% CI)		Relative effect (95% CI)	No of Participants (studies)	Quality of the evidence (GRADE)	Comments
	Assumed risk Control	Corresponding risk 5-FU				
**recurrence**	**Study population**		**OR 0.77** (0.07 to 8.7)	280 (2 studies)	⊕⊖⊖⊖ **very low**[1,2,3,4]	
	**229 per 1000**	**186 per 1000** (20 to 720)				
	**Moderate**					
	**279 per 1000**	**230 per 1000** (26 to 771)				

*The basis for the **assumed risk** (e.g. the median control group risk across studies) is provided in footnotes. The **corresponding risk** (and its 95% confidence interval) is based on the assumed risk in the comparison group and the **relative effect** of the intervention (and its 95% CI).

CI: Confidence interval; **OR:** Odds ratio;

GRADE Working Group grades of evidence
High quality: Further research is very unlikely to change our confidence in the estimate of effect.
Moderate quality: Further research is likely to have an important impact on our confidence in the estimate of effect and may change the estimate.
Low quality: Further research is very likely to have an important impact on our confidence in the estimate of effect and is likely to change the estimate.
Very low quality: We are very uncertain about the estimate.

1 Studies lacked details about allocation concealment and blinding
2 Event=51, less than 300
3 Heterogeneity: $Tau^2 = 2.74$; $Chi^2 = 9.69$, df = 1 (P = 0.002); $I^2 = 90\%$
4 OR (95%CI): 0.77 [0.07, 8.70]

D

β-RT for pterygium excision

Patient or population: patients with pterygium excision
Settings:
Intervention: β-RT

Outcomes	Illustrative comparative risks* (95% CI)		Relative effect (95% CI)	No of Participants (studies)	Quality of the evidence (GRADE)	Comments
	Assumed risk Control	Corresponding risk Β-RT				
**recurrence**	**Study population**		**OR 0.12** (0.01 to 1.03)	194 (2 studies)	⊕⊖⊖⊖ **very low**[1,2,3]	
	**458 per 1000**	**92 per 1000** (8 to 466)				
	**Moderate**					
	**492 per 1000**	**104 per 1000** (10 to 499)				

*The basis for the **assumed risk** (e.g. the median control group risk across studies) is provided in footnotes. The **corresponding risk** (and its 95% confidence interval) is based on the assumed risk in the comparison group and the **relative effect** of the intervention (and its 95% CI).

CI: Confidence interval; **OR:** Odds ratio;

GRADE Working Group grades of evidence
High quality: Further research is very unlikely to change our confidence in the estimate of effect.
Moderate quality: Further research is likely to have an important impact on our confidence in the estimate of effect and may change the estimate.
Low quality: Further research is very likely to have an important impact on our confidence in the estimate of effect and is likely to change the estimate.
Very low quality: We are very uncertain about the estimate.

1 Studies lacked details about allocation concealment and blinding
2 Heterogeneity: $Tau^2 = 2.05$; $Chi^2 = 6.99$, df = 1 (P = 0.008); $I^2 = 86\%$
3 Event=54, less than 300
