# Supplementary figures and images for: Anti-fibrotic, anti-VEGF or radiotherapy treatments as adjuvants for pterygium excision: a systematic review and network meta-analysis
Source: BMC Ophthalmol. 2017 Nov 25;17:211. doi: 10.1186/s12886-017-0601-5 (PMC5702200; doi:10.1186/s12886-017-0601-5)

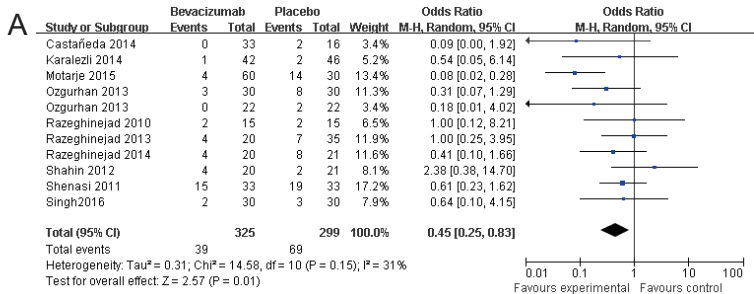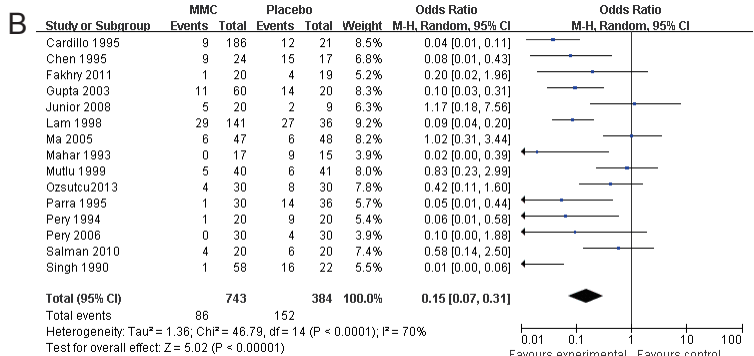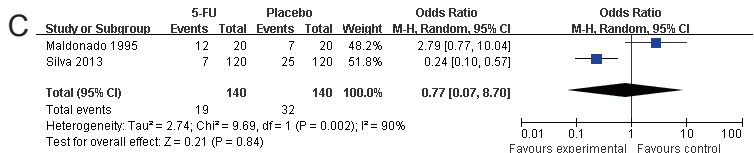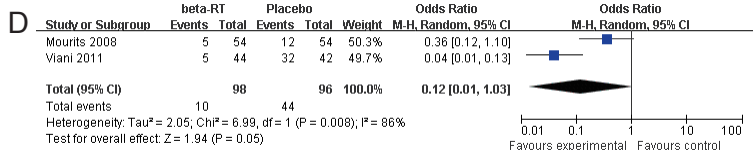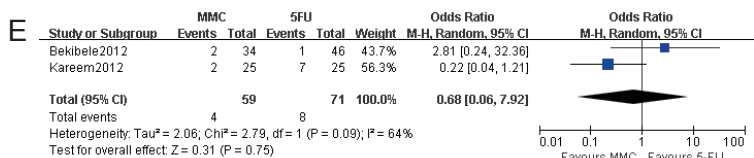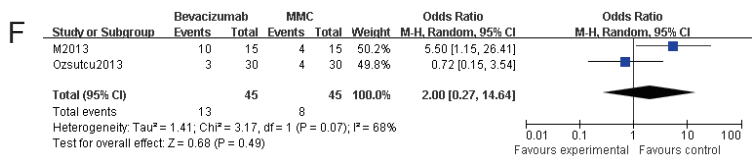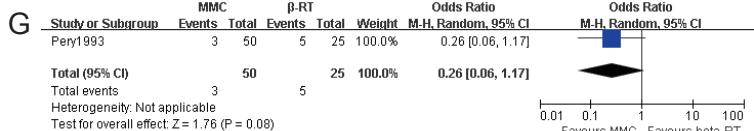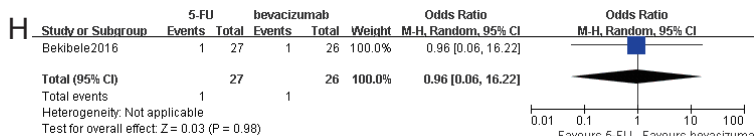

Supplement: Supplementary file 3 — Forest plots from the direct meta-analysis of recurrence between comparisons. Data are presented with odds ratios and 95% confidence intervals (CI) (PDF 385 kb) [file 12886_2017_601_MOESM3_ESM.pdf]
